# Genetic Addiction Risk and Psychological Profiling Analyses for “Preaddiction” Severity Index

**DOI:** 10.3390/jpm12111772

**Published:** 2022-10-27

**Authors:** Kenneth Blum, David Han, Abdalla Bowirrat, Bernard William Downs, Debasis Bagchi, Panayotis K. Thanos, David Baron, Eric R. Braverman, Catherine A. Dennen, Ashim Gupta, Igor Elman, Rajendra D. Badgaiyan, Luis Llanos-Gomez, Jag Khalsa, Debmalya Barh, Thomas McLaughlin, Mark S. Gold

**Affiliations:** 1 Division of Addiction Research & Education, Center for Sports, Exercise, and Mental Health, Western University of Health Sciences, Pomona, CA 91766, USA; 2 Division of Nutrigenomics, The Kenneth Blum Behavioral Neurogenetic Institute, LLC, Austin, TX 78701, USA; 3 Institute of Psychology, ELTE Eötvös Loránd University, 1075 Budapest, Hungary; 4 Department of Psychiatry, University of Vermont, Burlington, VT 05405, USA; 5 Department of Psychiatry, Wright University Boonshoft School of Medicine, Dayton, OH 45324, USA; 6 Division of Nutrigenomics, Victory Nutrition International, Inc., Harleysville, PA 19329, USA; 7 Centre for Genomics and Applied Gene Technology, Institute of Integrative Omics and Applied Biotechnology, Nonakuri, Purba Medinipur 721172, West Bengal, India; 8 Department of Molecular Biology, Adelson School of Medicine, Ariel University, Ariel 40700, Israel; 9 Department of Management Science and Statistics, University of Texas at San Antonio, San Antonio, TX 78249, USA; 10 Department of Pharmaceutical Sciences, College of Pharmacy, Southern University, Houston, TX 77004, USA; 11 Behavioral Neuropharmacology and Neuroimaging Laboratory on Addictions, Clinical Research Institute on Addictions, Department of Pharmacology and Toxicology, Jacobs School of Medicine and Biosciences, State University of New York at Buffalo, Buffalo, NY 14260, USA; 12 Department of Psychology, State University of New York at Buffalo, Buffalo, NY 14260, USA; 13 Department of Family Medicine, Jefferson Health Northeast, Philadelphia, PA 19107, USA; 14 Future Biologics, Lawrenceville, GA 30043, USA; 15 Department of Psychiatry, Harvard School of Medicine, Cambridge, MA 02115, USA; 16 Department of Psychiatry, South Texas Veteran Health Care System, Audie L. Murphy Memorial VA Hospital, Long School of Medicine, University of Texas Health Science Center, San Antonio, TX 78229, USA; 17 Department of Psychiatry, MT. Sinai School of Medicine, New York, NY 10003, USA; 18 Department of Microbiology, Immunology and Tropical Medicine, School of Medicine, George Washington University, Washington, DC 20052, USA; 19 Medical Consequences of Drug Abuse and Infections Branch, National Institute on Drug Abuse, NIH, Bethesda, MD 20892, USA; 20 Department of Genetics, Ecology and Evolution, Institute of Biological Sciences, Federal University of Minas Gerais, Belo Horizonte 31270-901, Brazil; 21 Department of Psychiatry, Washington University School of Medicine, St. Louis, MO 63110, USA

**Keywords:** genetic addiction risk analysis, reward deficiency syndrome (RDS), behavioral octopus, neurobiology, epigenetics, dopamine homeostasis, preaddiction

## Abstract

Since 1990, when our laboratory published the association of the *DRD2 Taq A1* allele and severe alcoholism in JAMA, there has been an explosion of genetic candidate association studies, including genome-wide association studies (GWAS). To develop an accurate test to help identify those at risk for at least alcohol use disorder (AUD), a subset of reward deficiency syndrome (RDS), Blum’s group developed the genetic addiction risk severity (GARS) test, consisting of ten genes and eleven associated risk alleles. In order to statistically validate the selection of these risk alleles measured by GARS, we applied strict analysis to studies that investigated the association of each polymorphism with AUD or AUD-related conditions, including pain and even bariatric surgery, as a predictor of severe vulnerability to unwanted addictive behaviors, published since 1990 until now. This analysis calculated the Hardy–Weinberg Equilibrium of each polymorphism in cases and controls. Pearson’s χ^2^ test or Fisher’s exact test was applied to compare the gender, genotype, and allele distribution if available. The statistical analyses found the OR, 95% CI for OR, and the post risk for 8% estimation of the population’s alcoholism prevalence revealed a significant detection. Prior to these results, the United States and European patents on a ten gene panel and eleven risk alleles have been issued. In the face of the new construct of the “preaddiction” model, similar to “prediabetes”, the genetic addiction risk analysis might provide one solution missing in the treatment and prevention of the neurological disorder known as RDS.

## 1. Background

The purpose of this article is to provide the scientific basis for the potential incorporation of genetic addiction risk (DNA polymorphic alleles) and psychological profiling analyses for an emerging novel concept referred to as the “preaddiction” severity index, as encouraged by both the National Institute on Drug Abuse (NIDA) and the National Institute on Alcohol Abuse and Alcoholism (NIAAA).

Addiction scientists and clinicians face an incredible challenge in combatting the current opioid and alcohol use disorder (OUD/AUD) pandemic worldwide. Despite significant progress, the death toll from narcotic overdoses managed to reach over 100,000 fatalities in the United States in 2021 and could reach 165,000 in 2022. NIDA and NIAAA continue to struggle with the generation of novel approaches to combat the severity of the current substance abuse epidemic. FDA-approved medication-assisted treatments (MAT) work primarily by blocking dopamine release and function at the pre-neuron in the nucleus accumbens [1,2]. Although MAT has reduced overdose deaths, costs, and healthcare events, a long-term strategy to return MAT patients to premorbid functioning is necessary. Medication-assisted treatments routinely fail [3], and when discontinued, relapse and overdose occur at rates similar to those of untreated patients. Neurologically, MAT may induce persistent changes that compromise endorphin, dopamine, and multiple brain systems. Chronic use of agonist therapies may be necessary for lack of other options; however, we caution that data on chronic vs. acute use harm reduction is lacking [4,5]. However, there is evidence that treatments themselves, such as long-term agonist treatments for OUD, may also cause reward deficiency syndrome (RDS) [5], causing harm and fatal consequences that eclipse the size of the current viral epidemic.

While the highest in the United States, drug overdose deaths are a problem internationally, and extraordinary solutions are needed. Short-term opioid substitution therapy can reduce harm. However, long-term patients can be locked into a lifetime of substance use disorders (SUD) [5]. Alternatively, inducing “psychological extinction” by weakening a conditioned response over time using the narcotic antagonist, Naltrexone, which blocks delta and Mu opioid receptors [6]. However, one difficulty encountered when using narcotic antagonism is compliance, which is moderated by the individual’s genetic antecedents [7]. The other approaches approved by the FDA for alcoholism block dopaminergic signaling [8,9]. 

There is increasing movement to opt for the non-addicting narcotic antagonist Naltrexone to treat Alcohol Use Disorder (AUD). Recent studies have shown that Naltrexone is beneficial by attenuating craving via “psychological extinction” and reducing relapse. Buprenorphine is currently the MAT of choice, but injectable Naltrexone plus an agent to improve dopaminergic function and tone may renew interest amongst addiction physicians and patients. Even with the extended injectable option, there is still poor compliance. As such, our group described an open-label investigation in humans showing improvement in naltrexone compliance and outcomes with dopamine augmentation with the pro-dopamine regulator KB220 (262 days) compared to naltrexone alone (37 days) [6]. This well-studied complex consists of amino-acid neurotransmitter precursors and enkephalinase-inhibitor therapy compared to treatment as usual. The consideration of this novel paradigm shift may assist in addressing not only the current opioid and alcohol epidemics but also the broader question of reward deficiency in general.

Understanding the above presupposition and emerging acceptance of the underlying concept of reward deficiency syndrome (RDS), now in ENCLYCLOPEDIA.COM, which Blum first conceived in 1995, facilitates the common mechanism hypothesis for chemical and behavioral addictions [10]. The typical neuromodulating aspects of neurotransmission and its disruption from chronic exposure to drugs and behavioral addictions necessitate an approach that involves attaining “dopamine homeostasis,” especially for AUD [11]. 

Subsequent large-scale genomics studies have had limited success in identifying the alleles implicated in addiction and RDS. Although genome-wide association studies (GWAS) and next-generation sequencing are valuable genetic tools, some fundamental issues exist. Certainly, GWAS, for example, helps to identify novel clusters of genes that may relate to an etiological component as a genetic antecedent to specific RDS behaviors, such as AUD. The next critical step following GWAS results is subsequent convergence to individual candidate genes. Thus, if there is indeed a clue or blueprint as to a specific known gene and associated polymorphic risk allele linked to specific phenotypes such as AUD, although the contribution of each gene may be small, it is indeed a worthwhile pursuit.

**Figure 1:**
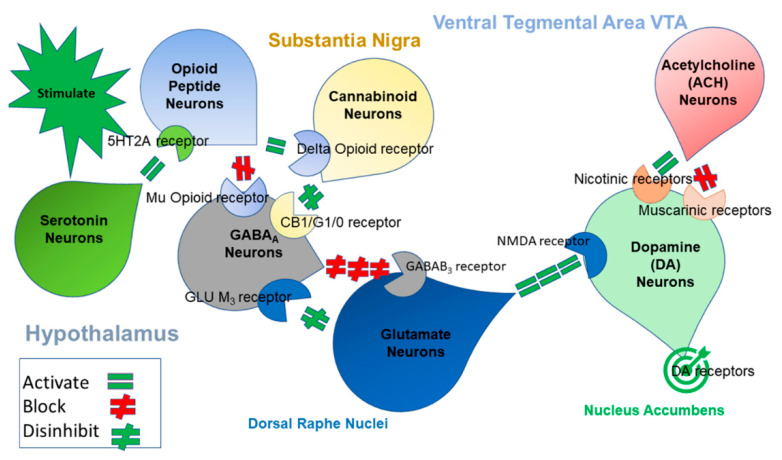
Illustrates the interaction of at least six major neurotransmitter pathways involved in the brain reward cascade (BRC). In the hypothalamus, environmental stimulation causes the release of serotonin, which in turn, via, for example, 5HT-2a receptors, activates (the green, equal sign), the subsequent release of opioid peptides into the hypothalamus. Then, the opioid peptides have two distinct effects, possibly via two different opioid receptors. (A) inhibits (the red hash sign) through the mu-opioid receptor (possibly via enkephalin) and projects to the substantia nigra to GABA_A_ neurons. (B) stimulates (the green, equal sign) cannabinoid neurons (e.g., anandamide and 2-archydonoglcerol) through beta-endorphin linked delta receptors, which in turn inhibit GABA_A_ neurons at the substantia nigra. cannabinoids, primarily 2-archydonoglcerol, when activated, can also indirectly disinhibit (the red hash sign) GABA_A_ neurons in the substantia nigra through activation of G1/0 coupled to CB1 receptors. Similarly, glutamate neurons located in the dorsal raphe nuclei (DRN) can indirectly disinhibit GABA_A_ neurons in the substantia nigra by activating GLU M3 receptors (the red hash sign). GABA_A_ neurons, when stimulated, will, in turn, powerfully (the red hash signs) inhibit ventral tegmental area (VTA) glutaminergic drive via GABAB_3_ neurons. Finally, glutamate neurons in the VTA will project to dopamine neurons through NMDA receptors (the green, equal sign) to preferentially release dopamine at the NAc shown as a bullseye indicating well-being (with permission Blum et al. [2]).

Several neurotransmitters are involved in the processing of reward and punishment. These pathways involve at least seven quintessential neurotransmitters and many second messengers linked to the mesolimbic and pre-frontal cortex (PFC). One function is to regulate the final pathway of “wanting,” causing net neuronal dopamine release. Figure 1 provides a schematic representation of the brain reward cascade (BRC), showing the interaction of serotonergic, cannabinoidergic, opioidergic, GABAergic, glutaminergic, acetylcholinergic, and dopaminergic systems related to net dopamine release at the nucleus accumbens (NAc). We highlight dopamine based on the understanding that healthy processing of an initial action potential in the brain requires the integrity of the entire neurotransmitter complex of the brain reward circuitry. The cascading interactions result in the balanced release of dopamine at the NAc and across many brain regions. These regions are involved in motivation, cognition (memory), pleasure, stress reduction, decision-making, recall, drug reinstatement, cravings, and well-being. 

## 2. Reward Deficiency Syndrome Index (RDSI) and Genetics 

Co-occurrences, similarities in the phenomenological and behavioral appearance, and empirical studies of some shared psychological and molecular mechanisms of addictive behaviors indicate a more integrative approach to the concept of addictive behaviors. The point of a number of studies was to scrutinize the possible genetic overlaps between different types of substance use, behavioral addictions, and other compulsive behaviors. A genetic association analysis was carried out as a part of the PGA study [12,13], assessing various kinds of addictions in a sample of 3003 adolescent participants. The genetic association analyses targeted 32 single-nucleotide polymorphisms (SNPs) and four substances of abuse (alcohol, tobacco, marijuana, and other drugs), and seven potentially addictive behaviors: internet addiction, gaming, social networking sites addiction, gambling disorder, exercise addiction, trichotillomania, and eating disorders. The association analysis revealed 29 nominally significant associations, of which nine survived the FDRbl correction for multiple testing. Four out of these nine significant associations were observed between a *FOXN3* SNP and various addictions: *rs759364* showed an association with the frequency of alcohol consumption and the mean scores of internet use, gaming, and exercise addiction questionnaires. In addition, significant associations have been found between *glial cell derived neurotrophic factor (GDNF) rs1549250, rs2973033, Cannabinoid Receptor-1* (*CNR1*) *rs806380*, and *Dopamine receptor D2* (*DRD2*)/*ankyrin repeat and kinase domain containing 1* (*ANKK1*) *rs1800497* variants and the “lifetime other drugs” variable.

In recent years, increasing quantities of scientific research have emphasized overlapping factors between the symptomatology of different types of addiction. Theoretical models of addictive disorders handling ‘addictions’ as a common disorder instead of distinct conditions have already been proposed in the 1980s [14]. More recent research also encourages considering addiction not as a collection of [15] different disorders but as a symbolic umbrella under which all addiction types can be classified. Additionally, Hollander’s obsessive-compulsive spectrum disorder (OCSD) model suggests a shared obsessive–compulsive spectrum in the background of psychiatric diseases of different diagnostic categories [15,16]. Furthermore, Blum, Cull, Braverman, and Comings in 1996 [17] proposed in the Reward Deficiency Syndrome that the development of impulsive and addictive behaviors shares some common psychological and molecular pathways. Focusing on the phenomenological aspects, the Component Model of Addictions by Griffiths [17] argues that all addictions share six essential characteristics. Empirical studies underlie the concept of shared psychological and molecular mechanisms proposed in these models. For example, tolerance is one of the critical criteria for many types of addiction. It takes a higher dose of the substance or behavior to achieve the same effect as earlier. Even a gambler can experience physical symptoms similar to opioid, stimulant, or polysubstance withdrawal [18,19,20]. Additionally, impaired social, occupational, or recreational ability, along with withdrawal symptoms, cravings, and unsuccessful quitting attempts, are critical elements in many addictions [21]. 

The classification and diagnostic criteria of addictive behaviors in the DSM-5 (APA, 2013) and the ICD-11 (WHO, 2018) also reflect these behaviors’ phenomenological similarities. The revised DSM-5 includes the updated category of “substance-related and addictive disorders,” replacing the former “substance-related disorders” category from the DSM-IV-TR. Although only gambling disorder is included in the DSM-V (under the non-substance related disorders category of substance-related and addictive disorders), the new “substance-related and addictive disorders” terminology in the DSM-V is much more permissive in regard to behavioral addictions. The same trend can be seen in ICD-11, in which gambling and gaming disorders were both included under the classification of psychiatric disorders [22].

The results of family, twin, and adoption studies estimate that the heritability (i.e., the overall genetic contribution) of addictions varies across the continent. Specifically, analyses revealed a significant single-nucleotide polymorphism-based heritability of 17 percent (SE = 5) in European ancestry (EAs) and 24 percent (SE = 15) in African ancestry (AAAs). Further, a significant genetic correlation of 0.77 (SE = 0.46) suggests that the allelic architecture influencing the alcohol dependence (AD) factor for Europeans and Africans is largely similar across the two populations. Analyses indicated that investigating the genetic underpinnings of alcohol dependence in different ethnic groups may serve to highlight the core etiological factors common to both groups as well as unique etiological factors specific to each ethnic group [23].

The brain’s reward system has a significant impact on behavioral control and plays a vital role in the pathophysiology of addictive behaviors. Dopaminergic and serotonergic neurotransmitter systems involved in these reward pathways are at the center of attention in candidate gene studies of substance abuse, non-substance addictions, and various risk-behavior-related traits, such as novelty seeking, impulsivity, or aggressive behavior (see reviews from our group [24,25] and others [26,27]).

In an attempt to resolve the controversy regarding the causal contributions of mesolimbic dopamine (DA) systems to reward, we evaluate the three main competing explanatory categories: “liking,” “learning,” and “wanting” [28]. That is, DA may mediate (a) the hedonic impact of rewards (liking), (b) learned predictions about rewarding effects (learning), or (c) the pursuit of rewards by attributing incentive salience to reward-related stimuli (wanting). We evaluate these hypotheses, especially as they relate to the RDS, and we find that the incentive salience, or “wanting” hypothesis of the DA function is supported by a majority of the evidence. Neuroimaging studies have shown that drugs of abuse, palatable foods, and anticipated behaviors such as sex and gaming affect brain regions involving reward circuitry and may not be unidirectional. Drugs of abuse enhance DA signaling and sensitize mesolimbic mechanisms that evolved to attribute incentive salience to rewards. Addictive drugs have in common that they are voluntarily self-administered, they enhance (directly or indirectly) dopaminergic synaptic function in the nucleus accumbens (NAc), and they stimulate the functioning of brain reward circuitry (producing the “high” that drug users seek). Although originally believed simply to encode the set point of hedonic tone, these circuits are now believed to be functionally more complex, also encoding attention, reward expectancy, disconfirmation of reward expectancy, and incentive motivation. Elevated stress levels, together with polymorphisms of dopaminergic genes and other neurotransmitter genetic variants, may have a cumulative effect on vulnerability to addiction. 

The RDS model of etiology holds very well for a variety of chemical and behavioral addictions. Interestingly, these concepts have been put to the test by Demetrovics and colleagues [29]. The aim of their investigation was to examine the dissociation between “wanting” and “liking” as a function of usage frequency, intensity, and subjective severity in individuals across four substances (alcohol, nicotine, cannabis, and other drugs) and ten behaviors (gambling, overeating, gaming, pornography use, sex, social media use, Internet use, TV-series watching, shopping, and work). Additionally, the potential roles of impulsivity and reward deficiency were investigated in “wanting,” “liking,” and well-being. The sex differences between “wanting” and “liking” were also examined. Based on our findings using structural equation modeling with 749 participants (503 women, *M_age_* = 35.7 years, *SD* = 11.84) who completed self-report questionnaires, “wanting” increased with the severity, frequency, and intensity of potentially problematic use, while “liking” did not change. Impulsivity positively predicted “wanting,” and “wanting” positively predicted problem uses/behaviors. Reward deficiency positively predicted problem uses/behaviors, and both impulsivity and problem uses/behaviors negatively predicted well-being. Finally, women showed higher levels of “wanting” compared to men. These findings demonstrate the potential role of incentive sensitization in both potentially problematic substance use and behaviors.

Functional neuroimaging studies have shown that cocaine, money, and beauty similarly energize the reward circuitry of the brain [30,31]. This suggests that, regardless of the object of the addiction, similar neurobiological pathways are stimulated in the brain [32]. Dopamine is released in the nucleus accumbens during rewarding experiences and reinforces the motivation for such activities. In addition, serotonin modulates the reward pathway and is involved in emotion and behavior regulation as well as consciousness [33]. Genetic factors may also influence how and when individuals develop addictive behaviors by affecting the sensitivity to drug effects at first use, reinforcing or inhibiting subsequent experimentation, and not just DSM diagnosis [34,35]. One of the universally accepted hypotheses in the development of addiction is RDS [36], which postulates that decreased dopamine receptor density or sensitivity in the brain causes a weaker reward sensation, and to compensate for it, individuals develop various types of behaviors to increase their dopamine levels. The syndrome consists of compulsive, addictive, and impulsive behaviors (e.g., drug addiction, compulsive eating, smoking, gambling, sex addiction, and internet gaming).

Although the dopamine system has been implicated as one of the most potent force influencers in the development and maintenance of addiction, dopamine itself does not function in isolation. The effects and net neuronal release of dopamine are influenced by several factors, involving a cascade of events (Figure 1), including the brain receptor system variability and other neurotransmitter systems (norepinephrine, GABA, glutamate, serotonin), which can be affected by various psychoactive substances and behaviors, e.g., opiates act through the endogenous opioid system, while alcohol through the GABA system, and RDS is indeed the actual phenotype [37]. Overall, neurobiological research indicates that different substances and behaviors stimulate remarkably similar neurobiological pathways [38].

The genetic and environmental overlap between different types of substance and behavioral addictions has been investigated [39]. Additionally, twin and other studies also concluded that the majority of the shared genetic and environmental factors are not substance-specific [40,41,42]. Identified genetic variants in addiction-related genes, e.g., *aldehyde dehydrogenases (ALDHs), gamma-aminobutyric acid receptor subunit alpha-2 (GABRA2)*, and *DRD2/ANKK1*, were strongly associated with dependence to various substances [43]. Gene network analysis showed immune signaling *and extracellular signal-regulated protein kinases 1* and *2* (*ERK1/2*) as novel genetic markers for multiple addiction phenotypes, including alcohol, smoking, and opioid addiction [44]. Cocaine and alcohol addition, as well as compulsive running, share common molecular pathways of substance and behavioral addictions [45,46]. The shared genetic vulnerability of pathological gambling and SUDs, primarily alcohol dependence, is another widely known example [47]. Twin research observed genetic overlap between substance use and gambling frequency [48]. Genome-wide studies indicated further evidence for a genetic association between pathological gambling and alcohol dependence [49]. These common genetic risk factors are also implicated in personality traits such as risk-taking, which are genetically associated with alcohol and drug use [50]. All these research results suggest that specific genetic markers generally increase the likelihood of addiction, regardless of the type of addiction.

In addition, pharmacological studies proved that even treatment non-specificity occurs between different substances, suggesting the same common neurobiological pathways in the background. Among these studies, naltrexone, an opioid antagonist used in opioid replacement therapy, was approved by the FDA to treat ethanol dependence [51,52,53]. Naltrexone has also proven to be efficient in the treatment of pathological gambling [54,55]. A partial nicotine agonist or even nicotine used for nicotine dependence has been used successfully in AUD [56], and methadone (an opioid agonist) has shown efficacy in reducing cocaine abuse among opioid-dependent patients [57]. The theory of addiction as a substance-independent disease is further supported by the perception that people recovering from a given substance commonly tend to change to another substance (e.g., from opiates to cocaine, alcohol, gambling) before successfully recovering from addiction [58]. Similar examples have been found for illicit drugs and nicotine, alcohol abuse and eating disorders, substance abuse, and pathological gambling. In fact, a systematic review synthesized the literature examining addiction substitution during recovery from substance use or behavioral addictions. A total of 96 studies were included, with sample sizes ranging from 6 to 14,885. The most common recovery addictions were opioids (30.21%), followed by cannabis (20.83%), unspecified use (17.71%), nicotine (12.50%), alcohol (12.50%), cocaine (4.17%), and gambling (2.08%). Statistical results were provided by 70.83% of the studies. Of these, 17.65% found support for addiction substitution, whereas 52.94% found support for concurrent recovery. A total of 19.12% found no statistical changes, and 10.29% found both significant increases and decreases. The remaining 29.17% of studies provided descriptive data without statistical tests. Predictors of addiction substitution were provided by 22.92% of the studies, and 11.46% included information on the impact of addiction substitution on treatment outcomes [59].

Growing evidence indicates overlaps between addictions: regardless of the nature of a specific activity, whether someone plays video games, gambles, uses the internet, or uses social networking sites, the extensive, repetitive, and problematic engagement in the above-mentioned activities might share similarities with SUDs. Co-occurrences and comorbidities are well-known in specific phenotypes, as many people with an addiction-related diagnosis have more than one psychiatric diagnosis [39,60]. The co-occurrence of chemical and behavioral addictions is well documented [61,62,63].

The co-occurrences of different types of addictive behaviors, similarities in their phenomenological and behavioral appearance, and empirical studies of some shared psychological and molecular mechanisms indicate a more integrative approach to the theoretical and nosological concept of addictive behaviors. This Hungarian study aimed to investigate the possible genetic overlaps between different types of substance use, behavioral addictions, and other compulsive behaviors, using a large sample from the psychological and genetic factors of addictions (PGA) study. The present genetic association analysis was carried out between 32 addiction-related candidate SNP and a wide range of substance use (nicotine, alcohol, marijuana, and other drugs) and potentially addictive behaviors (internet use, gaming, social networking site use, gambling, exercising, trichotillomania, and eating disorders). 

Analysis showed a significant association between *DRD2/ANKK1 rs1800497* and marijuana consumption at least four times during the past 30 days (Chi-square = 6.424; df = 1; *p* = 0.0113, OR = 0.613). The association showed that the minor allele (A) is more frequent among the users (27.1%) as compared to the never users (18.6%). It was also found a significant association between eating problems and *DRD4 rs1800955* [F(1, 4972) = 9.184, *p* = 0.0025, η^2^ = 0.002, power = 0.858, Cohen’s d = 0.09], where the minor allele was in association with lower mean scores on the EAI questionnaire (0.67 ± 0.92) as compared to the major (T) allele (0.76 ± 0.98) [39].

Previous genetic association studies of addictions have mainly focused on one specific type of SUD. The Hungarian study [39] aimed to investigate a broad spectrum of substance and non-substance addictions in a large sample consisting of high school and university students to find possible associations. The analyzed genes and polymorphisms were selected in part based on earlier literature results (e.g., nicotinic acetylcholine receptor gene clusters implicated by earlier GWAS studies [64,65] and partly because novel genetic targets have also been considered, including the GARS test [66].

In addition, the presented genetic association analysis was carried out as a part of the PGA study assessing several types of addictions in a sample of 3003 adolescent participants. As previously stated, the association analyses of 32 SNPs and four substance use and seven potentially addictive behaviors revealed 29 nominally significant associations, nine of which survived the FDRbl correction for multiple testing. Polymorphisms in the *CNR1* gene (rs806380) and the *DRD2/ANKK1* gene (rs1800497) also showed association with the “lifetime other drugs” variable. *CNR1* codes for cannabinoid 1 receptors that offer high expression in regions with proven involvement in reward, addiction, and cognitive function [67]. The *rs806380* SNP in intron 2 of *CNR1* is associated with cannabis dependence. However, the majority of the participants also met the criteria for alcohol dependence. The *rs806380* SNP has also been linked to the development of cannabis dependence symptoms [68]. A microsatellite polymorphism of *CNR1* has also been positively associated with intravenous drug use and cocaine, amphetamine, and cannabis dependence [69]. 

The *rs1800497* SNP of *ANKK1*, also known as the *Taq1A* polymorphism, has been widely studied in psychiatric disorders. Numerous studies have linked *Taq1A* to reduced *DRD2* densities and binding affinity [70,71], implying a direct or indirect influence on the dopamine concentration in the synaptic clefts. In addition, the *Taq1A* polymorphism has been significantly associated with nicotine dependence [72,73], smoking cessation [74], alcohol dependence [75,76], heroin dependence [77,78], and cocaine dependence [79,80].

Finally, the results revealed an association between *rs1800955* of the *DRD4* gene and the mean scores on the SCOFF questionnaire assessing eating disorders. This SNP, also known as -521C/T, is a variant in the promoter region upstream of the *DRD4* gene and has a putative role in regulating transcriptional activity [81]. Furthermore, in another study, additional haplotype analysis showed a significant association with a four-locus haplotype, including *rs1800955* [82]. Previous associations have been reported between alleles at -521C/T and novelty seeking, extraversion, and drug abuse [83,84]. The *rs1800955* polymorphism of *DRD4* has also been proposed in the GARS test by Blum et al. [85], which identifies alleles known to convey vulnerability to addiction and creates an assessment of the degree of vulnerability of an individual to develop addictive behavior [85]. This SNP and the *DRD2 A1* allele (rs1800497) have been associated with heroin addiction [86] and contribute to a “risk-taking phenotype” [87].

These alleles were proposed for a GARS panel in case–control studies, specifically for alcoholism (Table 1) [see ref. [88] for further explanation]. 

To develop this patented GARS test, the ten reward candidate genes selected included the dopamine receptors (*DRD1, 2, 3, 4*), *DAT1*, *SLC6A4*, *COMT*, *MAO-*A, *GABRB3*, *OPRM1*, and some SNPs and point mutations chosen to reflect a hypo dopaminergic trait. The genes determined to negatively influence the net release of dopamine at the brain reward site were chosen from thousands of association studies providing clear evidence of the specific risks for all addictions [89].

## 3. Understanding GARS

The BRC involves the interaction of genes and neurotransmitters and their control of the release of dopamine (Figure 1). Functional differences within the BRC, which could be genetic or epigenetic, may predispose individuals to addictive behaviors and altered pain tolerance [90,91]. The GARS test is the first United States/European patented test clinically proven to predict vulnerability to pain and various other addictive and compulsive behaviors identified as RDS. 

Strategies to combat the opioid epidemic of prescription drug misuse and death and the implication of dopaminergic tone in pain pathways have been proposed previously [92,93]. The site of a predisposition to pain sensitivity may be the mesolimbic projection system, where genetic variations are associated with pain vulnerability or tolerance [93]. These variations may provide specific targets to assist in the treatment of pain and identify risks for subsequent addiction. For example. many known gene variants are involved in opioid pharmacology. Therefore, genetic testing of candidate genes such as *DRD1, 2, 3, 4, MOA-A, COMT, DAT1, SLC6A4, OPRM1*, and *GABRB3* might result in pharmacogenomics, personalized solutions, and improved clinical outcomes. Identifying those within compromised populations at genetic risk for RDS behaviors may be a frontline tool for better resource allocation in municipalities [17,66,94,95], especially in the criminal justice system. 

There is a natural sequence of neurotransmission that produces feelings of well-being (Figure 1). The cascade events, including the synthesis, vesicle storage, metabolism, release, and other neurotransmitter functions, are regulated by gene expression. Genetic testing of relevant variants can provide a window into an individual’s neurochemistry, assisting providers in formulating optimal treatment options.

The release of dopamine, the neurotransmitter responsible for motivation and stress reduction, is the neurological reward cascade’s functional endpoint. As a result, individuals who are genetically predisposed hypodopaminergia seek out substances and behaviors that will help them overcome this trait by activating mesolimbic brain dopaminergic centers [96,97]. Lacking balanced dopamine function, an individual may have anhedonia, lack a sense of well-being, and may have difficulty with craving pleasure, lack of motivation, and coping with stress. Psychoactive substances and risky behaviors [98] induce DA release into the mesolimbic nucleus accumbens synapses to compensate for that individual’s hypo-dopaminergic trait/state [99].

Temporary relief from discomfort and a sense of well-being are the products of this self-medication, even in schizophrenia [100]. Pathological substance-seeking behaviors are employed to provide a pleasurable response and to decrease uncontrollable cravings. The chronic misuse of substances often leads to the inactivation, downregulation, and inhibition of neurotransmitter synthesis and neurotransmitter depletion, as observed in reward dysregulation [101]. Those individuals with risk-reward gene polymorphisms/variations, who experience environmental insults, will be at high risk for compulsive, impulsive, and addictive behaviors that are collectively referred to as RDS, a spectrum that includes and characterizes genetically induced behaviors, including anhedonia [102]. These pathological behaviors include addiction, tolerance, and dependence to chronic opioid use, licit or illicit. The behavior or drug chosen by the individual is a function of both genetic and environmental factors, such as the availability of the substance and peer pressure.

**Figure 2:**
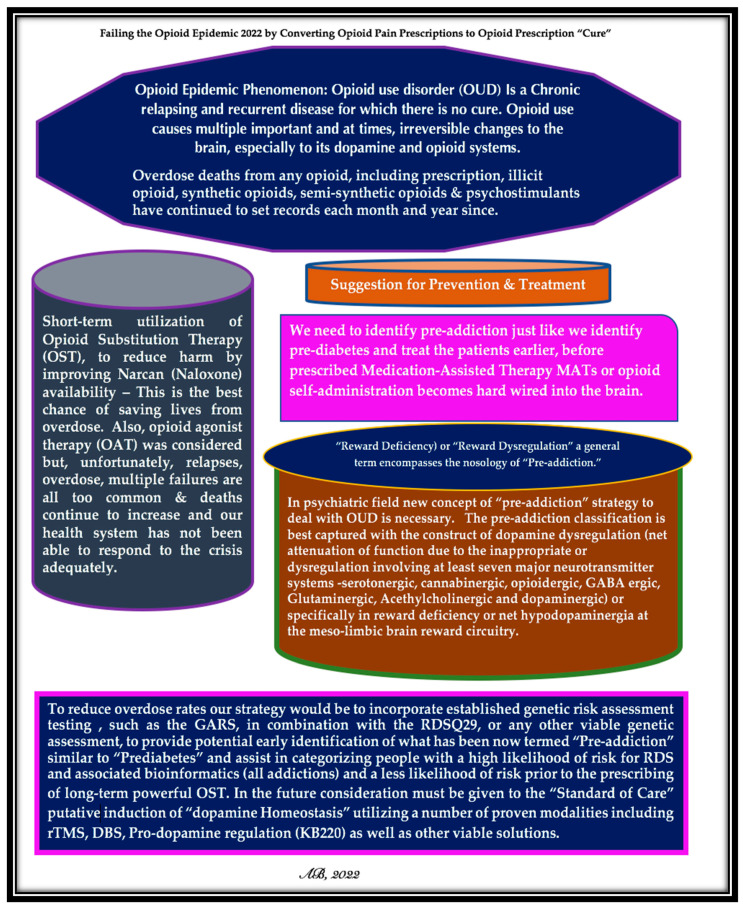
Schematic illustration linking the current drug abuse crisis and preaddiction.

Initially, eleven polymorphisms in ten genes selected for the development of GARS test are alleles that contributed most to the hypodopaminergic trait RDS and were chosen following an extensive literature review. The selection involved thousands of studies associated with alleles with significant risk for addictive behaviors, both drug and non-drug RDS (Figure 2). In previous research from Blum et al. [103] evaluating 273 mixed-gender patients attending seven treatment centers who completed the addiction severity index-media version V (ASI-MV), GARS significantly predicted drug severity (equal or >four alleles) and alcohol severity (equal or >seven alleles).

## 4. Initial DNA Customized Studies

Previously, our laboratory started with the mindset of designing a study with which to evaluate DNA customization with nutritional solutions for both wellness and especially weight management, as one RDS example. In terms of nutrigenomics, a number of studies should be mentioned to serve as the rationale for developing customized DNS-guided pro-dopamine regulation utilizing KB220 as a basis [104,105,106,107,108,109]. In a series of experiments, Blum’s laboratory genotyped 1058 subjects and administered KB220 (formerly LG8839, Recomposize, and Genotrim). KB220 is a neuroadaptogen nutraceutical that includes specific calibrated amounts of dl-phenylalanine, chromium, l-tyrosine, and other select amino acids and adaptogens based on polymorphic outcomes. The resultant customized formulae involved a minimum of 175 SNPs covering 16 genes relevant to the BRC in an attempt to induce “dopamine homeostasis.” Specifically, in this small cohort, using simple *t*-tests comparing many parameters before and after 80 days of consumption of KB220Z, we found significant positive changes in many vital parameters, including reduced weight and lower body mass index (BMI). It is noteworthy that only the *DRD2* gene polymorphism (Al allele) had a significant Pearson correlation using days on treatment (*r* = 0.42, *p* = 0.045). Importantly, this twofold increase is significant for compliance with treatment. Blum’s research team methodically assessed the impact of polymorphisms from five possible genes and their potential as targets for the development of a DNA-customized nutraceutical KB220Z to combat obesity, with particular emphasis on body re-composition as measured by BMI. Blum’s group developed an early version of the GARS specifically directed towards glucose craving and, as such, included specific alleles: *DRD2 Al; MTHFR C 677T; 5HT2a 1438G/A; PPAR-γProl2Ala* and *Leptin Ob1875* < 208 *bp*. Pre- and post-hoc analyses revealed a significant difference between the starting BMI and the BMI following an average of 41 days (28–70 days) of KB220Z intake in the 21 individuals. Additionally, the average pretreatment weight in pounds was 183.52 compared to the post-treatment weight of 179, a statistically significant (*p* < 0.047) change. In this particular group, 53% lost, on average, over 2.5% of their starting weight. These studies in obesity are presented to provide some idea of the potential success of utilizing customized nutrigenomic-based solutions, which may pave the way to treating and preventing RDS-like behaviors in the future [104,105,106,107,108,109]. 

## 5. Can Early Genetic Risk Assessment of “Preaddiction” like “Prediabetes” Provide the Missing Piece to Help Overcome Substance Use Disorder?

Unfortunately, despite the enormous efforts of the federal government to help fund and develop and deliver certain treatments (MAT) for victims of SUD, albeit not a magic bullet or “cure,” treatment penetration rates are less than 20% [110]. In an article by McLellan et al. [111], they correctly point out that the diabetes field faces a similar dilemma and were able to increase treatment penetration through early-stage diabetes identification, termed “prediabetic.” In fact, in 2001, the American Diabetes Association suggested that the term “prediabetic” could operationally be defined by augmented scores on two laboratory tests: impaired glucose tolerance and impaired fasting glucose [112]. This strategy led to a wide range of campaigns and partnerships with third-party payors and, over time, has shown increased risk detection rates, shortened delays between symptom onset and treatment entry, and success in halting progression to diabetes [113].

It is noteworthy that Volkow (Director of NIDA) and Koob (Director of NIAAA) are encouraging the psychiatric field to include the concept of “preaddiction” as a plausible new inclusion for the DSM. Relevant to this suggestion is the possibility of developing a test to help categorize mild, moderate, or high risk for future addictive-like behaviors. With this in mind, based on our initial work and now with many other global scientists, the preaddiction classification is best captured with the construct of dopamine dysregulation (net attenuation of function due to the inappropriate or dysregulation involving at least seven major neurotransmitter systems—serotonergic, cannabinergic, opioidergic, GABAergic, glutaminergic, acethylcholinergic, and dopaminergic) or specifically in reward deficiency or net hypodopaminergia at the meso–limbic brain reward circuitry [114]. Currently, there are 1449 articles listed in PUBMED (7/20/22), of which approximately 47% are independent of our laboratory, and 221 articles listed in PUBMED using the search term “Reward Deficiency Syndrome”. Our point here is that while the term “preaddiction” resonates well with the historical advances in the diabetic field, scientifically, the real evidence resides in concepts related to brain neurotransmitter deficits or even, in some cases, surfeit (especially in adolescence as a neurodevelopmental event) referred to as “reward dysregulation” [115]. It is noteworthy, as pointed out by McLellan et al. [111], that while the DSM-5 uses 11 equally weighted symptoms of impaired control to define SUDs along a three-stage severity continuum. The common name “addiction” is reserved for severe SUD, defined by six or more symptoms and found in approximately 4% to 5% of adults. Those with mild to moderate SUD (i.e., two to five symptoms) comprise a much larger proportion of the adult population (13%), and thus account for far more substance use–related harm to society than those with severe SUD (i.e., addiction). However, treatment efforts and public health policies have focused almost exclusively on those with serious, usually chronic addictions, virtually ignoring the much larger population with early-stage SUDs. Although harmful substance misuse and early-stage SUDs can be identified and severity progression monitored, little has been conducted, especially where it is most common, in mainstream healthcare settings. Indeed, neither clinicians nor the public even have a commonly understood name for early-stage SUD. In this regard, we are proposing “reward deficiency“ (meaning lack of normal function) or “reward dysregulation” as a general term that does encompass the nosology of “preaddiction.” In stating this suggestion, we are cognizant that for public awareness, the latter terminology would be more understood. However, for the DSM, psychiatrists, and other clinicians, the former seems more parsimonious [116]. 

Independent of the appropriate name, similar to the idea of “prediabetes,” we want to develop a reliable method that allows for the early identification of people at risk for future serious issues with substance and non-substance behavioral addictions (preaddiction). Therefore, we are hereby proposing the GARS test along with the RDSQ29 [117] pencil and paper test to capture the psychological correlates of RDS. In terms of GARS, albeit requiring additional research, there are 58 listed articles in PUBMED. Unfortunately, they are predominately from Blum’s laboratory and mostly narrative in content, but still encouraging. Importantly, there have been a number of studies published showing real utility and scientific benefit in terms of identifying both drug and alcohol risk by utilizing objective DNA polymorphic identification rather than just subjective (but still useful) diagnostic surveys that include family history [118]. To point out a few examples, there have been published works on a number of important clinical issues. 

The GARS test was utilized to assess the potential risk of preaddiction in patients who, following an injury, for example, received powerful opioids to relieve pain and continued to be prescribed an analgesic for more than a year. In this study, Moran et al. [94] utilized RT-PCR for SNP genotyping and multiplex PCR/capillary electrophoresis for fragment analysis of the role of eleven alleles in a ten-reward gene panel, reflecting the activity of brain reward circuitry in 121 chronic opioid users. The study consisted of 55 males and 66 females, averaging 54 and 53 years of age, respectively. The patients included Caucasians, African Americans, Hispanics, and Asians. The inclusion criteria mandated that the morphine milligram equivalent (MME) be 30–600 mg/day (males) and 20 to 180 mg/day (females) for the treatment of chronic pain over 12 months. Ninety-six percent carried four or more risk alleles, and 73% carried seven or more risk alleles, suggesting a high predictive risk for opioid and alcohol dependence, respectively.

In addition, an estimation based on these previous literature results provided herein, while not representative of all association studies known to date, this sampling of case-control studies displays significant associations between alcohol and drug risk. In fact, Blum et al. [119] presented a total of 110,241 cases and 122,525 controls derived from the current literature. While we may take argument concerning many of these so-called controls (e.g., blood donors), it is quite remarkable that there are a plethora of case–control studies indicating the selective association of these risk alleles (measured in GARS) for the most part, indicating hypodopaminergia. 

In another investigation, Fried et al. [120] utilized GARS on an entire family. Specifically, the proband was a female with a history of drug abuse and alcoholism. She experienced a car accident while under the influence and voluntarily entered treatment. Following an assessment, she was genotyped using the GARS and started a neuronutrient with a KB220 base indicated by the identified polymorphisms. She began taking it in April 2018 and continues. She had success in recovery from SUD and improvement in socialization, family, economic status, well-being, and attenuation of major depression disorder. She tested negative over the first two months of treatment and in a recent screening. After approximately two months, her parents also decided to take the GARS and started taking the recommended variants. The proband’s father (a binge drinker) and mother (no SUD) both showed improvement in various behavioral issues. Finally, the proband’s biological children were also GARS tested, showing a high risk for SUD. This three-generation case series represents an example of the impact of genetic information coupled with an appropriate DNA-guided “pro-dopamine regulator” in the recovery and enhancement of life.

The risk of all addictive drug and non-drug behaviors, especially in the unmyelinated PFC of adolescents, is important and complex. Many animal and human studies show the epigenetic impact on the developing brain in adolescents compared to adults. Some reveal an underlying hyperdopaminergia that seems to set our youth up for risky behaviors by inducing high quantities of pre-synaptic dopamine release at reward site neurons. In addition, altered reward gene expression in adolescents caused epigenetically by social defeat, like bullying, can continue into adulthood. In contrast, there is also evidence that epigenetic events can elicit adolescent hypodopaminergia. This complexity suggests that neuroscience cannot make a definitive claim that all adolescents carry a hyperdopaminergic trait. The primary issue involves the question of whether there exists a mixed hypo- or hyper-dopaminergia in this population. One investigation by Blum et al. [88] utilized GARS testing in 24 Caucasians ages 12–19 derived from families with RDS. It was found that adolescents from this cohort, derived from RDS parents, displayed a high risk for any addictive behavior (a hypodopaminergia), especially drug-seeking (95%) and alcohol-seeking (64%). The adolescents in our study, although more work is required, show a hypodopaminergic trait derived from a family with RDS. Certainly, in future studies, our laboratory will analyze GARS in non-RDS Caucasians between the ages of 12–19. The suggestion is first to identify risk alleles with the GARS test and then use well-researched precision pro-dopamine neutraceutical regulation. This “two-hit” approach might prevent tragic fatalities among adolescents, potentially with preaddiction, in the face of the American opioid/psychostimulant epidemic.

Since 1990, when our laboratory published the association of the *DRD2 Taq A1* allele and severe alcoholism in *JAMA*, there has been an explosion of genetic candidate association studies, including GWAS. To develop an accurate test to help identify those at risk for at least AUD, Blum’s group [88] applied strict analysis to studies that investigated the association of each polymorphism with AUD or AUD-related conditions published from 1990 until 2021. This analysis calculated the Hardy–Weinberg Equilibrium of each polymorphism in cases and controls. Pearson’s *χ*^2^ test or Fisher’s exact test was applied to compare the gender, genotype, and allele distribution if available. The statistical analyses found the OR, 95% CI for OR, and the post risk for 8% estimation the population’s alcoholism prevalence revealed a significant detection. The OR results showed significance for *DRD2, DRD3, DRD4, DAT1, COMT, OPRM1*, and *5HTT* at 5%. While most of the research related to GARS is derived from our laboratory, we are encouraging more independent research to confirm our findings.

Inquiry into the neurobiology of addictive disorders has consistently pointed to the altered brain mesolimbic dopaminergic and other neurotransmitter circuits subserving reward and motivation. Even in the era of GWAS, the SUD field remains uniform. However, given the uncertain genetic- vs. acquired nature of the observed alterations. To that end, a multi-center study utilized GARS criterion validity against the backdrop of the ASI-MV in 393 polydrug abusers. Blum and associates [121] found a significant relationship between GARS and the ASI-MV alcohol severity score. While those with high drug severity likewise had heightened GARS, this association was not linear. Sequence variation in multiple genes regulating dopaminergic signaling influenced risk in an additive manner; age was a significant covariate. Higher numbers (≥7) of reward-gene-polymorphisms that moderate reduced dopamine signaling were significantly associated with higher ASI-MV alcohol severity scores. Whereas higher numbers (≥4) reward-gene-polymorphisms that moderate reduced dopamine signaling were significantly associated with higher ASI-MV drug severity scores. Our results replicate those of prior reports implicating dopamine in the course of alcoholism and drug abuse and extend these prior findings by suggesting a preexisting GARS-defined polygenic risk factor that may be modulated by age-related pathophysiological and environmental variables. Further studies are needed to investigate the corresponding endophenotypes, particularly the involvement of the RDS stemming from the hypofunctional dopaminergic system [121].

Finally, to reiterate our understanding of the daunting polygenicity of mental illness, Hyman [122] discussed this perplexing issue. A momentous opportunity to elucidate the pathogenic mechanisms of psychiatric disorders has emerged from advances in genomic technology, new computational tools, and the growth of international consortia committed to data sharing. Moreover, as espoused by Hyman [81], the resulting large-scale, unbiased genetic studies have yielded new biological insights and, with them, the hope that a half-century of stasis in psychiatric therapeutics will come to an end. However, we agree that “a sobering picture is coming into view; it reveals daunting genetic and phenotypic complexity, portending enormous challenges for neurobiology.”

Additionally, the successful exploitation of results from genetics will require past avoidance of long-successful reductionist approaches to the investigation of gene function, a commitment to supplanting much research now conducted in model organisms with human biology, and the development of new experimental systems and computational models to analyze polygenic causal influences. In this regard, our laboratory has already started the process by developing behavioral addiction risk assessment (BARS/GARS) testing in psychiatry [73,74,75,76,77,78,79,80]. Furthermore, psychiatric neuroscience must develop a new scientific map to guide investigation through a polygenic “*terra incognita*” [122] and a reconsideration of what constitutes the real brain map. In our view, while finding new and novel GWAS discovered clusters of genes are incredibly important to determining the genetic risk for RDS, it is also prudent to consider finite candidate genes involved in the dynamic systems biological approach of at least the major neurotransmitter pathways, as portrayed in the well-known BRC (Figure 1).

The idea of preaddiction, which was first introduced in 1971 and thus not a new term [123], while a potentially smart idea, the concept espoused by McLellan et al. [111] is fraught with some misjudgments. Most recently, Yatan Pal Singh Balhara, from the National Drug Dependence Treatment Center and Department of Psychiatry, All India Institute of Medical Sciences (AIIMS), New Delhi, India, commented. The authors, McLellan et al. [111], make an argument for the introduction of the concept of preaddiction. They also propose that the existing categories of mild and moderate SUDs in DSM-5 can be used to operationalize “preaddiction” in the interim. Two points that highlight the limitation and challenges of this approach to operationalization. First, there was a significant shift in the way disorders, were due to the use of psychoactive substances, were being diagnosed by the introduction of the diagnostic category of “SUDs” in the DSM-5.2. The terms “abuse,” “dependence,” and “addiction” were not used in the DSM-5. Additionally, the severity of the SUD was assessed based on the number of diagnostic criteria (out of a total of 11) that were met. The DSM-5 continuum of the severity of SUD does not demarcate those ‘without addiction’ (commonly equated with “mild” and “moderate” severity categories) from those with “addiction” (commonly equated with “severe” category). Some of the core features of the concept of “addiction” can be present even in those with mild and moderate severity of SUD. For example, in the case where a person uses a substance in a pattern that is characterized by “substance being taken in larger amounts over a longer period than was intended; a persistent desire or unsuccessful efforts to cut down or control substance use; recurrent substance use resulting in a failure to fulfill major job obligations; tolerance; and withdrawal,” the severity rating in such a case shall be moderate. This presentation would fit into the conceptualization of “addiction,” and using the term ‘preaddiction’ in such a case would fail to capture the clinical presentation accurately. 

In fact, there can be clinical presentations where a lesser number of criteria are present, but these criteria are indicative of the presence of “addiction.” Second, the clinical presentations that are captured by mild and moderate severity are given a valid medical diagnosis as per the DSM-5. This should warrant appropriate clinical interventions (brief intervention, laboratory investigations, promotion of health and well-being, prevention of progression, treatment, disability limitation, rehabilitation-focused, recovery-oriented, etc.). If the aim of the introduction of the concept of “preaddiction” is to offer appropriate interventions to those at risk of developing “addiction” later in life, then these individuals need to be identified using criteria that do not overlap with an existing diagnostic category [124]. 

To be clear, in fact, RDS occurs at birth due to DNA genetic antecedent risk, which could set up individuals to be at high risk, which is actually equivalent to preaddiction. Of course, via epigenetic insults due to subsequent SUD, the polymorphic DNA antecedents could compound the risk. 

To assist the readership, we developed Figure 2 as a schematic linking the current drug abuse crisis and preaddiction. 

## 6. Conclusions

Since 1990, when our laboratory published the association of the *DRD2 Taq A1* allele and severe alcoholism in JAMA, there has been an explosion of genetic candidate association studies, including GWAS. To develop an accurate test to help identify those at risk for at least AUD, a subset of RDS, Blum’s group developed the GARS test, consisting of ten genes and eleven associated risk alleles. In order to statistically validate the selection of these risk alleles measured by GARS, we applied strict analysis to studies that investigated the association of each polymorphism with AUD or AUD-related conditions, including pain and even bariatric surgery, as a predictor of severe vulnerability to unwanted addictive behaviors, published since 1990 until now. This analysis calculated the Hardy–Weinberg Equilibrium of each polymorphism in cases and controls. Pearson’s χ^2^ test or Fisher’s exact test was applied to compare the gender, genotype, and allele distribution if available. The statistical analyses found the OR, 95% CI for OR, and the post risk for 8% estimation of the population’s alcoholism prevalence revealed a significant detection [88]. It is relevant that while the GARS test does not actually stratify low, median, or high preaddiction status, the newly developed RDSQ29, which has been validated [117], could be utilized in combination with the GARS.

Prior to these results, the United States and European patents on a ten-gene panel and eleven risk alleles have been issued. In the face of the new construct of the “preaddiction” model, similar to “prediabetes”, the genetic addiction risk analysis might provide one solution missing in the treatment and prevention of the neurological disorder known as RDS.

## Figures and Tables

**Table 1:** Gene polymorphisms under consideration and the literature summary.

Gene/Polymorphism	Number of Studies	Overall Summary
*Dopamine D1 Receptor (DRD1):* *rs4532—risk allele G*	3	Several studies supported that genetic variation in *DRD1* may influence genetic predisposition to alcoholism. A statistically significant association of *DRD1 rs4532* polymorphism with alcohol dependence was found among Indian males (90 cases vs. 122 controls). Other studies also demonstrated that this could be associated with the impulsivity and aggression of AUD patients.
*Dopamine D2 Receptor (DRD2): rs1800497—risk allele A1*	118	The *DRD2 rs1800497* was found to be associated with the risk of AUD and several AUD-related conditions in a meta-analysis of numerous case–control studies (total of 18,290 cases vs. 19,809 controls, including US Caucasian, native and African American, British, French, Italian, Swedish, Finnish, Spanish, Mexican, Brazilian, Scandinavian, and Japanese) pooled with the random effect models.
*Dopamine D3 Receptor (DRD3): rs6280—risk allele C (Ser9Gly)*	3	Several case–control studies investigated the association between the *DRD3 rs6280* polymorphism and alcohol dependence. In a Korean study (243 cases vs. 130 controls), the *DRD3 rs6280* polymorphism was significantly associated with AUD development.
*Dopamine D4 Receptor (DRD4): rs1800955—risk allele C (48bp repeat VNTR)*	35	The *DRD4 rs1800955* polymorphism was found to be associated with the risk of developing AUD and AUD-related conditions in a meta-analysis of various case–control studies (total of 2997 cases vs. 2588 controls, including US Caucasian, Mexican American, and Indian) pooled with the random effect models.
*Dopamine Transporter Receptor (DAT1): SLC6A3 3’-UTR—risk allele A9 (40bp repeat VNTR)*	43	The central dopaminergic reward pathway is likely involved in alcohol intake and the progression of alcohol dependence. *DAT1* is a principal regulator of dopaminergic neurotransmission. From the meta-analysis of numerous case–control studies (total of 3790 cases vs. 3446 controls) pooled with the random effect models, the *DAT1 SLC6A3* 3’-UTR risk allele was found to be marginally associated with the risk of AUD and AUD-related conditions.
*Catechol-O-Methyltransferase (COMT): rs4680—risk allele G (Val158Met)*	13	*COMT* is a strong candidate gene that contributes to SUD and schizophrenia. A meta-analysis of several case–control studies (total of 1212 cases vs. 933 controls, including US Caucasian, Finnish, Croatian, and Taiwanese) pooled with a random effect model, the association of *COMPT rs4680* polymorphism with the risk of developing AUD and AUD-related conditions was found with marginal statistical significance.
*µ-Opioid Receptor (OPRM1): rs1799971—risk allele G (A118G)*	28	Opioid receptors play an essential role in ethanol reinforcement and alcohol dependence risk. Polymorphisms in the *OPRM1* gene expressing µ-opioid receptors could be significantly associated with some features of alcohol dependence. From the meta-analysis of case–control studies (total of 3096 cases vs. 2896 controls, including US Caucasian, Spanish, Turkish, and Asian), pooled with the random effect model, the results indicated that a functional *OPRM* variant is associated with the risk of alcohol dependence with marginal statistical significance.
*γ-Aminobutyric Acid (GABA) A Receptor, β-3 Subunit (GABRB3): CA repeat—risk allele 181*	6	The GABAergic system has been implicated in alcohol-related behaviors. From case–control studies (171 cases vs. 45 controls), the association of variants of the *GABRB3* gene with alcohol dependence is, however, inconclusive. A more extensive controlled study is required for improved results.
*Monoamine Oxidase A (MAO-A): 3’ 30bp VNTR -risk allele 4R DNRP*	6	The function of monoamine oxidase (*MAO*) in alcoholism was determined using several case–control studies (170 cases vs. 177 controls). Although genetic heterogeneity is suspected of underlying alcoholism and *MAO-A* mutations may play a role in susceptibility to alcoholism, the overall results were not found to be statistically significant. A more extensive controlled study is required to obtain conclusive results.
*Serotonin Transporter Receptor (5HTT) Linked Promoter Region (5HTTLPR) in SLC6A4: rs25531—risk allele S’*	20	Serotonin (5-HT) has been demonstrated to regulate alcohol consumption. Since the activity of the 5-HT transporter protein (*5-HTT*) regulates 5-HT levels, it may contribute to the risk of alcohol dependence. A meta-analysis of case–control studies (total 9996 cases vs. 9950 controls) pooled with the random effect models showed a significant association between alcohol dependence and the serotonin-transporter-linked promoter region (*5-HTTLPR)*, which is a polymorphic region in the *SLC6A4* gene.

## Data Availability

Data are contained within the manuscript.
